# Spread of Psoriasiform Inflammation to Remote Tissues Is Restricted by the Atypical Chemokine Receptor ACKR2

**DOI:** 10.1016/j.jid.2016.07.039

**Published:** 2017-01

**Authors:** Kave Shams, Gillian J. Wilson, Mark Singh, Ellen H. van den Bogaard, Michelle L. Le Brocq, Susan Holmes, Joost Schalkwijk, A. David Burden, Clive S. McKimmie, Gerard J. Graham

**Affiliations:** 1 Chemokine Research Group, Institute of Infection, Immunity and Inflammation, 120 University Place, University of Glasgow, Glasgow, UK; 2 Department of Dermatology, Radboud Institute for Molecular Life Sciences (RIMLS), Radboud University Medical Center, Nijmegen, The Netherlands; 3 Glasgow Royal Infirmary, 84 Castle Street, Glasgow, UK; 4 Department of Dermatology, Lauriston Building, Edinburgh, UK; 5 Virus Host Interaction Team, Leeds Institute of Cancer and Pathology, University of Leeds, St James’ University Hospital, Leeds, UK

**Keywords:** ACKR2, atypical chemokine receptor 2, IMQ, imiquimod, PASI, Psoriasis Area Severity Index, WT, wild type

## Abstract

Elucidating the poorly defined mechanisms by which inflammatory lesions are spatially restricted in vivo is of critical importance in understanding skin disease. Chemokines are the principal regulators of leukocyte migration and are essential in the initiation and maintenance of inflammation. The membrane-bound psoriasis-associated atypical chemokine receptor 2 (ACKR2) binds, internalizes and degrades most proinflammatory CC-chemokines. Here we investigate the role of ACKR2 in limiting the spread of cutaneous psoriasiform inflammation to sites that are remote from the primary lesion. Circulating factors capable of regulating ACKR2 function at remote sites were identified and examined using a combination of clinical samples, relevant primary human cell cultures, in vitro migration assays, and the imiquimod-induced model of psoriasiform skin inflammation. Localized inflammation and IFN-γ together up-regulate ACKR2 in remote tissues, protecting them from the spread of inflammation. ACKR2 controls inflammatory T-cell chemotaxis and positioning within the skin, preventing an epidermal influx that is associated with lesion development. Our results have important implications for our understanding of how spatial restriction is imposed on the spread of inflammatory lesions and highlight systemic ACKR2 induction as a therapeutic strategy in the treatment and prevention of psoriasis and potentially a broad range of other immune-mediated diseases.

## Introduction

Chemokines, the primary in vivo regulators of leukocyte migration, are central to inflammatory pathogenesis (Rot and von Andrian, 2004, Zlotnik and Yoshie, 2000). They bind to 7-transmembrane spanning receptors to orchestrate the recruitment of inflammatory cells into and within tissues. In addition to the classical signaling chemokine receptors (Bachelerie et al., 2014a), there exists a subfamily of stromally expressed atypical chemokine receptors that display promiscuous ligand binding and atypical signaling responses after ligand binding (Bachelerie et al., 2014b, Graham et al., 2012, Nibbs and Graham, 2013). These atypical chemokine receptors can function as chemokine scavengers, thereby fine-tuning or limiting chemokine responses in vivo. One of these molecules, atypical chemokine receptor 2 (ACKR2; previously known as D6), is a high-affinity receptor for multiple inflammatory CC-chemokines (Graham and Locati, 2013). ACKR2 does not mount classical signaling responses after ligand binding (Borroni et al., 2013) but internalizes ligands and targets them for intracellular degradation (Weber et al., 2004). ACKR2 has therefore been characterized as a scavenging receptor for inflammatory CC-chemokines. ACKR2’s role in limiting the development of inflammation is shown by numerous studies of ACKR2-deficient mice and by reports of aberrant expression patterns in human inflammatory pathologies (Bazzan et al., 2012, Cochain et al., 2012, Codullo et al., 2011, Singh et al., 2012).

Recently, we showed that ACKR2 expression is particularly elevated in uninvolved skin of psoriatic patients (Singh et al., 2012). We hypothesized that inflammatory products from primary inflamed lesions limit the spread of chemokine-dependent inflammation by driving systemic up-regulation of ACKR2 at remote sites. Here, we present experimental in vitro and in vivo validation of, and a mechanistic explanation for, our hypothesis. We highlight circulating products of the primary inflamed site, notably T-helper (Th) 1/Th17 cytokines, as inducers of remote ACKR2 expression. We show an anti-inflammatory role for increased ACKR2 expression at remote noninflamed sites in vivo and provide a mechanism whereby focal inflammation reduces the propensity for inflammation to spread to remote tissues through modulation of ACKR2. Overall, we propose that dynamic regulation of ACKR2 expression is an endogenous means for limiting spread of psoriasiform inflammation.

## Results

### ACKR2-deficient mice display exaggerated inflammation in response to imiquimod

**Figure 1:**
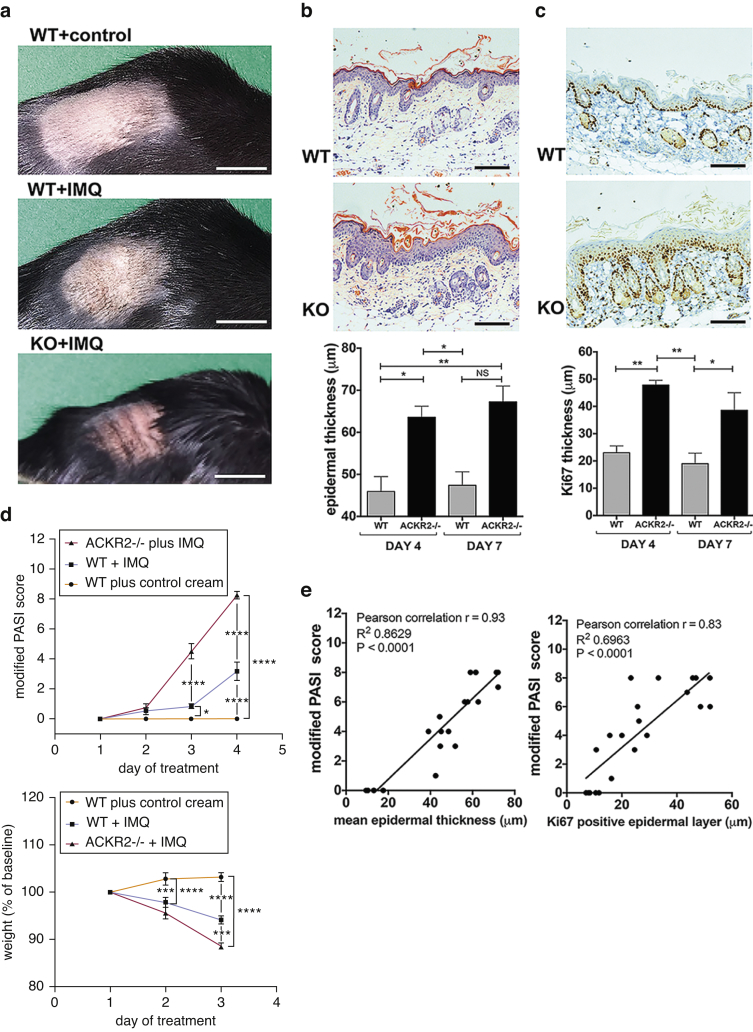
**ACKR2-deficient mice display an exaggerated psoriasiform phenotype in response to IMQ treatment.** (**a**) WT mice treated for 3 days (culled day 4) with (top) vehicle control and (middle) IMQ; (bottom) IMQ-treated ACKR2-deficient mice. Scale bars = 1 cm. (**b**) Hematoxylin and eosin-stained WT and ACKR2-deficient (KO) skin after 3 days of IMQ treatment and quantification of epidermal thickness after 3 or 6 days of daily IMQ. Scale bars = 100 μm. Statistics: one-way analysis of variance. (**c**) Ki67-stained WT and ACKR2-deficient (KO) skin after 3 days of IMQ and quantification of thickness of Ki67 staining in the epidermis. (**d**) (top) PASI skin inflammation and (bottom) weight change (baseline = 100%) throughout treatments. Statistics: two-way analysis of variance, Tukey’s multiple comparisons test. (**e**) Correlation between mean epidermal thickness and PASI score (left). Correlation between mean thickness of Ki67 positive epidermis and PASI score (right). ^∗^*P* < 0.05, ^∗∗^*P* < 0.01. ACKR2, atypical chemokine receptor 2; IMQ, imiquimod; KO, knockout; NS, not significant; PASI, Psoriasis Area Severity Index; WT, wild type.

To establish a mouse model appropriate for assessing the mechanism of differential ACKR2 regulation and the role of ACKR2 in regulating localized inflammation, we determined the role of ACKR2 in the well-characterized imiquimod (IMQ) mouse model of psoriasis (van der Fits et al., 2009). At rest, there are no histological differences in wild-type (WT) and ACKR2-deficent skin (Baldwin et al., 2013, Jamieson et al., 2005, Nibbs et al., 2007). However, in response to daily topical IMQ application (Aldara cream, Meda Pharmaceuticals, Bishops Stortford, UK) and compared with WT mice, ACKR2-deficient mice displayed an exaggerated cutaneous inflammatory response (Figure 1a). This was associated with significantly increased epidermal thickness (Figure 1b, and see Supplementary Figure S1a online) and keratinocyte proliferation (Figure 1c, and see Supplementary Figure S1b). The dynamics of psoriasis-like pathology during induction of inflammation was quantified using a modified Psoriasis Area Severity Index (PASI) (see Supplementary Figure S2a online), which showed that ACKR2-deficient mice rapidly develop substantially worse pathology than WT mice (Figure 1d). This was accompanied by significantly more weight loss in ACKR2-deficient mice (Figure 1d). The PASI score correlated directly with the extent of epidermal thickness and keratinocyte proliferation, which were both significantly enhanced in ACKR2-deficient mice (Figure 1e). Together, these data show that ACKR2 is required for limiting inflammation in a relevant in vivo model of psoriasis.

### ACKR2 regulates chemokine-mediated T-cell migration to control the positioning of CD3^+^ T lymphocytes in vivo

**Figure 2:**
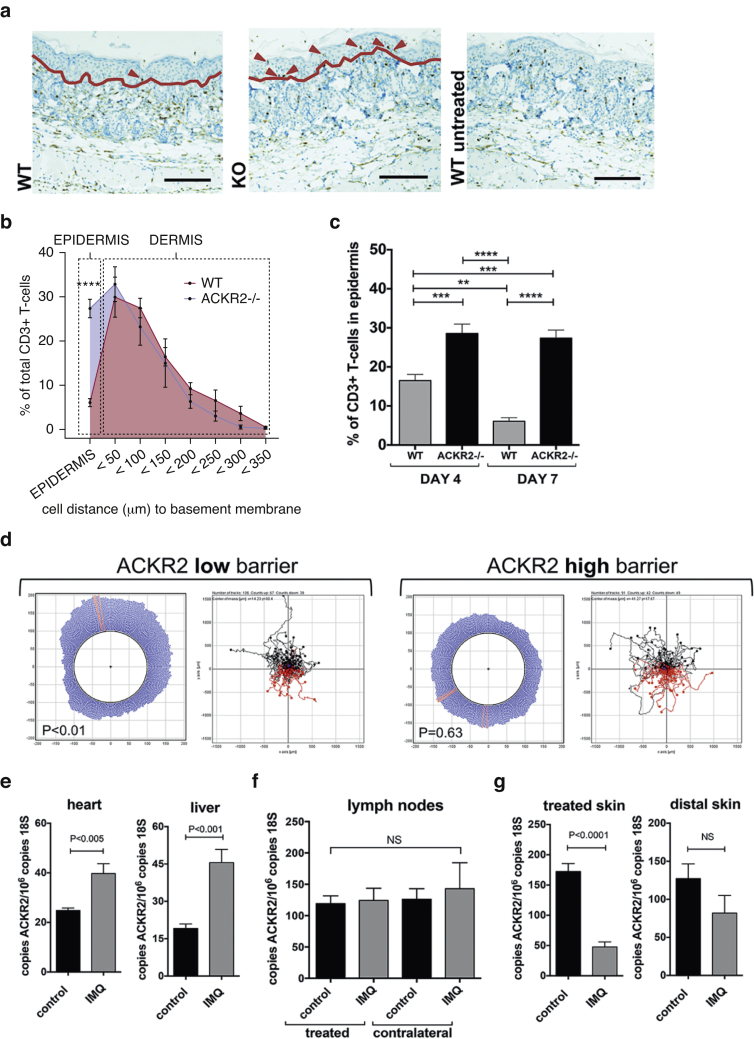
**ACKR2 regulates T-cell migration and positioning in the skin.** (**a**) CD3^+^ staining after 3 days of treatment with IMQ. Arrows: CD3+ T cells. Red line: dermal-epidermal junction. (**b**) Distance of CD3^+^ cells from basement membrane after 3 days of treatment with IMQ. Two-way analysis of variance, Sidak’s multiple comparisons test. (**c**) Epidermal CD3^+^ as percentage total CD3^+^ cells (one-way analysis of variance, Tukey’s multiple comparisons test). (**d**) T-cell migration toward CCL5 (top of plots) across low or high ACKR2-expressing keratinocytes. Plots on left: cumulative migration direction. Plots on right: individual tracks (black = toward and red = away from CCL5). Statistics: Rayleigh test. (**e**) ACKR2 expression in heart and liver from vehicle- (black bars) or IMQ- (grey bars) treated mice. (**f**) ACKR2 expression in draining lymph nodes from treated or untreated skin. Mice were treated 3 days with vehicle (black bars) or IMQ (grey bars). (**g**) ACKR2 at site of vehicle/IMQ treatment and distal dorsal skin. ^∗∗^*P* < 0.01, ^∗∗∗^*P* < 0.005, ^∗∗∗∗^*P* < 0.0001. ACKR2, atypical chemokine receptor 2; IMQ, imiquimod; KO, knockout; NS, not significant; WT, wild type.

T-cell migration into the epidermis is essential for the development of psoriasiform inflammation (Conrad et al., 2007). We therefore determined the effect of ACKR2 on CD3^+^ T-cell localization in the IMQ model. There are no basal differences in total cutaneous or epidermal T-cell numbers in ACKR2-deficient, compared with WT, mice (Baldwin et al., 2013, Jamieson et al., 2005). However, there was an increase in CD3^+^ T-cell numbers in skin of IMQ-inflamed WT and ACKR2-deficient mice compared with vehicle-treated mice (Figure 2a). Also, although T cells were, in the main, excluded from inflamed WT epidermis (Figure 2a–c), they were able to migrate efficiently into inflamed ACKR2-deficient epidermis, even in areas of epidermis overlying dermis that exhibited relatively few T cells (Figure 2a and see Supplementary Figure S3 online).

**Figure 3:**
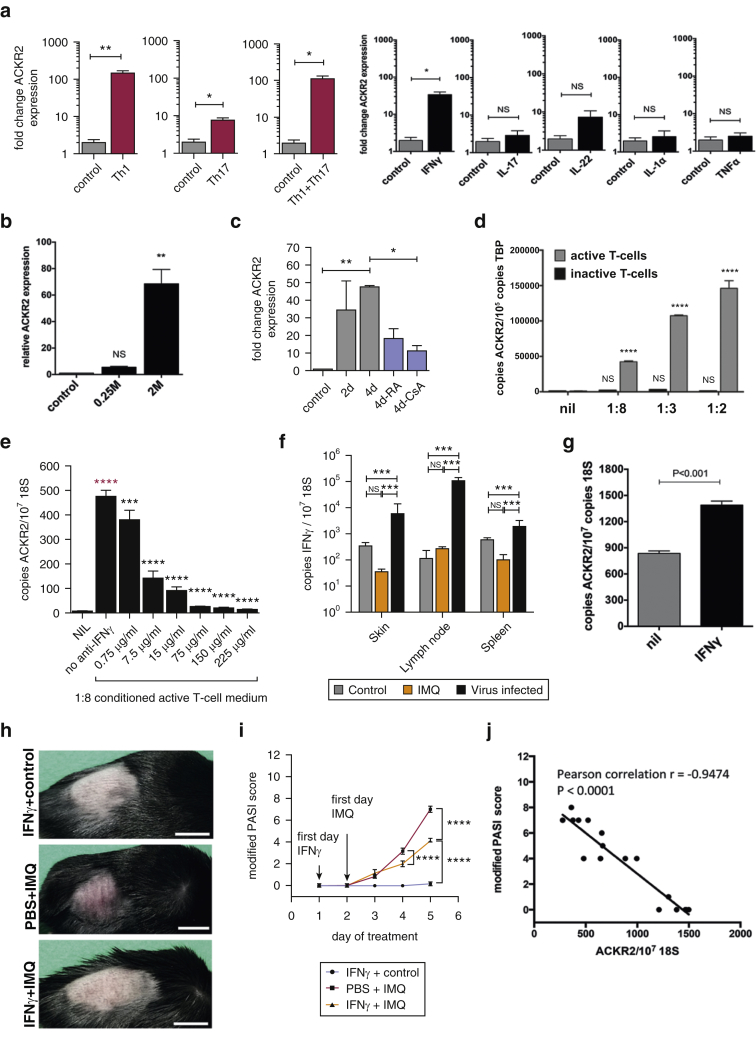
**Cytokines from activated T cells induce ACKR2 expression in human keratinocytes.** ACKR2 expression in response to cytokines (100ng/ml) in (**a**) human keratinocytes, (**b**) skin equivalents treated with activated CD4^+^ T cells and (**c**) skin equivalents after 2–4 days with/without treatment with all-*trans* retinoic acid or cyclosporine A. ACKR2 expression in (**d**) human keratinocytes treated with T-cell conditioned medium alone and (**e**) human keratinocytes treated with anti–IFN-γ antibodies. Two-way analysis of variance, Tukey’s MCT. (**f**) Mouse IFN-γ expression 24 hours after treatment. Virus-infected samples act as positive control. One-way analysis of variance, Tukey’s multiple comparisons test. (**g**) ACKR2 expression in mouse dorsal skin after IFN-γ treatment (20,000U/day for 4 days, n = 6). Student *t* test. (**h**) WT mice were treated for 3 days and photographed on day 4. Scale bars = 1 cm. (**i**) PASI score. Arrows: first day of IFN-γ/phosphate buffered saline injections and IMQ/vehicle. (**j**) Correlation of cutaneous ACKR2 expression and PASI score. ^∗^*P* < 0.05, ^∗∗^*P* < 0.01, ^∗∗∗^*P* < 0.005, ^∗∗∗∗^*P* < 0.0001. ACKR2, atypical chemokine receptor 2; CsA, cyclosporine A; d, days; IMQ, imiquimod; M, million; NS, not significant; PASI, Psoriasis Area Severity Index; RA, retinoic acid; TBP, TATA-binding protein; Th, t helper.

Next, to understand the mechanism by which ACKR2 controls T-cell localization in vivo, we tested whether ACKR2 expressed by a barrier layer of keratinocytes (as seen in the skin dermal-epidermal junction) could regulate human T-cell migration. For this purpose, we established an in vitro three-dimensional cell migration assay. The T-cell chemoattractant chemokine CCL20 has been associated with psoriasis (Lowes et al., 2014). However, CCL20 does not bind to ACKR2 (Madigan et al., 2010) and thus will not contribute to the altered T-cell localization. We therefore developed an assay using the ACKR2 ligand CCL5, which is highly expressed in human psoriatic plaques (Singh et al., 2012) and is a potent attractant for human T cells (Makino et al., 2002). CCL5 was placed at the top of a skin collagen matrix, below which we placed a barrier layer of primary human keratinocytes that separated the chemokine from the T cells at the bottom of the collagen-filled chamber (see Supplementary Figure S4 online). Cultured keratinocytes express low ACKR2 at rest but can be induced to express very high levels (see Figure 3a and c). Although a barrier of low-ACKR2-expressing keratinocytes had little influence on T-cell migration toward CCL5, high-ACKR2-expressing IFN-γ–stimulated keratinocytes effectively blocked directional migration of T cells toward the chemokine (Figure 2d). This indicates that the up-regulation of ACKR2 in the barrier layer of basal keratinocytes prevents CCL5-induced directional T-cell migration into the epidermis.

We next wanted to determine whether ACKR2 expression is differentially modulated in the in vivo IMQ model of psoriasis. We found that localized cutaneous inflammation was associated with significantly increased ACKR2 transcript expression in apparently uninvolved tissues located away from the primary site of inflammation, including heart and liver (Figures 2e). We noted no gross histological abnormalities in these tissues in either WT or ACKR2^–/–^ mice treated with or without IMQ (see Supplementary Figure S5 online). We refer to such sites (especially cutaneous sites) as being remote from the primary inflammatory lesion. In contrast, no differences were seen in ACKR2 transcript expression in lymph nodes draining either inflamed or noninflamed cutaneous sites (Figure 2f). In keeping with our data from human psoriasis (Singh et al., 2012), ACKR2 expression was reduced within psoriasis-like inflammatory lesions of IMQ-treated skin compared with noninflamed skin (Figure 2g). However, and in contrast to the data for heart and liver, no significant differences in cutaneous ACKR2 mRNA expression were seen in skin remote from the site of IMQ treatment (Figure 2g).

Together, our data show that ACKR2 determines localization of inflammatory T cells at sites of inflammation and that localized inflammation, induced by IMQ, leads to remote up-regulation of ACKR2 expression in noninflamed tissues.

### Th1/Th17 cytokines induce ACKR2 expression in epidermal keratinocytes and protect cutaneous sites from inflammation

Increased epidermal ACKR2 expression in human uninvolved psoriatic skin and psoriatic peripheral blood leukocytes (Singh et al., 2012) suggests regulation of expression by systemic circulating factors derived from inflammatory plaques. To gain insights into these factors in vivo we performed multiplex analysis on plasma from healthy control subjects and psoriatic patients. The most striking and significant differences in psoriatic patients were seen with levels of cytokines strongly associated with activated Th1 and Th17 phenotypes (see Supplementary Figure S6 online). We therefore tested whether Th1/Th17 cytokines could increase ACKR2 mRNA expression in healthy primary human keratinocytes. Treatment of keratinocytes with cytokine cocktails, representative of Th1 and Th17 cells, increased ACKR2 transcript expression (Figure 3a). In addition, and as previously reported (Singh et al., 2012), IFN-γ was active as a potent, single-agent inducer of ACKR2 mRNA expression.

We then examined how adding variable numbers of activated T-cells affected ACKR2 transcript expression in an in vitro human skin equivalent model of psoriasis (van den Bogaard et al., 2014). As shown (Figure 3b), activated T cells dose-dependently increased ACKR2 expression in this model, and the T-cell dependency of this effect was confirmed using cyclosporine A (Figure 3c). Next, we collected total conditioned medium from in vitro activated T cells, which markedly increase keratinocyte ACKR2 mRNA expression in a dose-dependent manner (Figure 3d). To assess the relative contribution of IFN-γ, tissue culture supernatant of activated human T cells was treated with anti–IFN-γ antibodies, which neutralized the potent ACKR2-inducing effect (Figure 3e). Because T-cell derived factors are major regulators of keratinocyte ACKR2 expression, we additionally assessed levels of these factors in the plasma of IMQ-treated mice. Our analysis showed that IFN-γ levels were below the limits of detection of the assays. In addition, we did not detect IFN-γ expression within the inflammatory lesions in IMQ-treated mice (Figure 3f). Thus, although the IMQ mouse model recapitulates key aspects of the cutaneous pathology of human psoriasis, it is not associated with elevated IFN-γ expression and does not recapitulate the high circulating IFN-γ levels observed in patients (see Supplementary Figure S6). We therefore systemically injected IFN-γ (as a prototypic cytokine with ACKR2-inducing properties to better model human disease) into mice, which led to a significant increase in ACKR2 expression in dorsal skin (Figure 3g).

We next wanted to determine if increased ACKR2 could protect mice from developing inflammation in response to IMQ treatment. Because IFN-γ is deficient in the IMQ model of psoriasis, and thus incompletely recapitulates human disease, IFN-γ was injected into mice twice daily throughout the IMQ treatment protocol, and the extent of inflammation was determined. As shown (Figure 3h), IFN-γ administration was associated with reduced signs of inflammation in WT mice after IMQ treatment and, using our modified PASI, this reduction was shown to be significant (Figure 3i). In addition, ELISA analyses showed no impact of IMQ or IFN-γ on IFN-γ expression within the inflamed skin (in keeping with Figure 3f) but showed up-regulation of the T-cell chemoattractant chemokines CCL5 and CCL20, which were blocked in response to systemic IFN-γ treatment (see Supplementary Figure S7 online). CCL20 protein expression was also induced in ACKR2-deficient mice after IMQ treatment (see Supplementary Figure S7), and this was also reduced by IFN-γ treatment. In keeping with the reported inhibitory effects of IFN-γ on Th17 cell differentiation (Harrington et al., 2005, Park et al., 2005), IL-17 levels were reduced in IMQ-inflamed skin in response to IFN-γ treatment (see Supplementary Figure S7). ACKR2 transcript levels in treated skin displayed a strong inverse correlation with the PASI, supporting the conclusion that systemic (IFN-γ–driven) increases in ACKR2 expression ameliorated cutaneous inflammatory pathology (Figure 3j).

### Remote cutaneous inflammation, together with IFN-γ, induces systemic up-regulation of ACKR2 and limits the spread of inflammation

**Figure 4:**
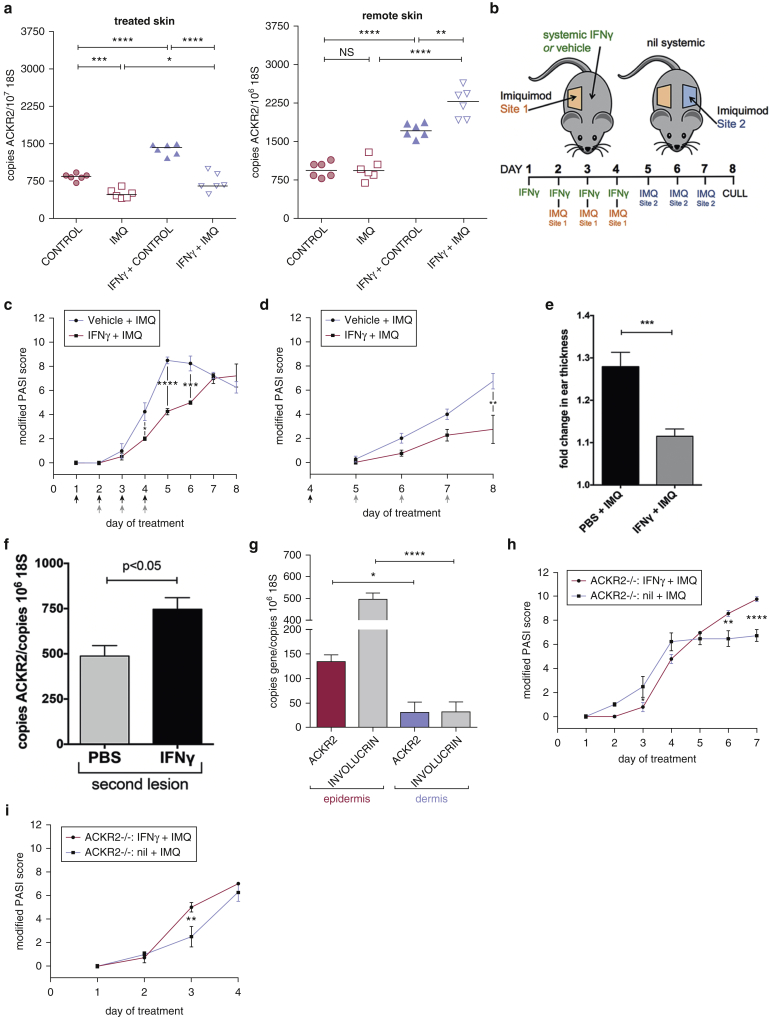
**Localized IMQ combined with systemic IFN-γ ameliorates psoriasis-like pathology at both primary and distal sites in an ACKR2-dependent manner.** (**a**) ACKR2 expression (day 5) in treated and distal/remote untreated skin of IMQ-treated mice (days 1–4) in presence/absence of systemic IFN-γ (20,000 U/day). Statistics: Student *t* test. (**b**) Experimental design. PASI score at (**c**) initial lesions and (**d**) lesion induced by IMQ at second site. (**e**) As in **c** and **d** with ear as second site. Ear thickness was measured in mice treated on the flank with IMQ and systemic IFN-γ (20,000 U/day) or PBS. (**f**) ACKR2 expression in second IMQ-induced lesions. First IMQ lesion was induced with concurrent PBS (grey bar) or IFN-γ (black bar). Statistics: Student *t* test. (**g**) ACKR2 expression in distal epidermis and dermis. Epidermal/dermal separation confirmed by involucrin quantitative PCR (grey bars). Statistics: Student *t* test. PASI score of IMQ-treated ACKR2-deficient mice pretreated with systemic PBS or IFN-γ (20,000 U/day) at (**h**) initial lesion and at (**i**) secondary lesion. Statistics: two-way analysis of variance. ^∗^*P* < 0.05, ^∗∗^*P* < 0.01, ^∗∗∗^*P* < 0.005, ^∗∗∗∗^*P* < 0.0001. ACKR2, atypical chemokine receptor 2; IMQ, imiquimod; NS, not significant; PASI, Psoriasis Area Severity Index; PBS, phosphate buffered saline.

To determine the effect of localized inflammation on remote cutaneous ACKR2 expression, we examined the impact of local IMQ treatment of skin with concurrent systemic IFN-γ administration on remote skin ACKR2 expression. Systemic administration of IFN-γ led to significant increases in cutaneous ACKR2 expression at the local site of both control cream and IMQ treatment (Figure 4a). Although IMQ treatment alone did not result in increased ACKR2 transcript levels at remote cutaneous sites (Figures 2g and 4a), IMQ combined with systemic IFN-γ significantly increased ACKR2 expression in remote skin to levels higher than those seen with IFN-γ alone (Figure 4a), thus successfully replicating the human psoriatic expression pattern of ACKR2. To examine the sustained impact of IFN-γ/IMQ up-regulation of remote ACKR2 expression on subsequent cutaneous inflammatory responses, we initially treated a primary skin site (right flank) with IMQ with concurrent systemic administration of vehicle control or IFN-γ. The IMQ and IFN-γ treatment was stopped at day 4, and from that point onward a second remote noninflamed cutaneous site (left flank) was treated with topical IMQ (experimental design summarized in Figure 4b). Note that mice receiving either vehicle or IFN-γ in the absence of IMQ treatment displayed no skin inflammation and had a PASI score of 0. These essentially negative data are not included in subsequent figures. As expected from the above data, systemic IFN-γ administration reduced pathology at the primary treatment site (Figure 4c), which plateaued at day 5 in the vehicle-treated mice but at day 8 in the IFN-γ–treated mice. Although we have no clear explanation for the increased PASI score in the IFN-γ–treated mice between days 5 and 8, these data may also indicate a role for IFN-γ in delaying the inflammatory process. After cessation of IFN-γ/IMQ at the primary site, subsequent IMQ-induced inflammation at the remote site (contralateral flank skin) was reduced in mice receiving both IMQ and IFN-γ for the initial 4 days (Figure 4d). We obtained similar data in which initial systemic IFN-γ in WT mice significantly reduced the inflammatory response to IMQ when using the ear as a distal site of inflammation (Figure 4e). Q-PCR showed that localized psoriasiform inflammation and IFN-γ coordinately up-regulated remote tissue ACKR2 transcript levels (Figure 4f) and that this occurred predominantly in the epidermal layer (Figure 4g). To confirm that the remote protective effect of systemic IFN-γ is mediated specifically by ACKR2, experiments were also conducted in ACKR2-deficient mice. As predicted, and in contrast to WT mice, IFN-γ did not confer a protective effect at either the initial (Figure 4h) or remote (Figure 4i) IMQ treatment sites in ACKR2-deficient mice. In fact, at some time points, IFN-γ administration exacerbated pathology, suggesting that in ACKR2-deficient mice with their inherent inability to regulate inflammatory CC-chemokine activity, IFN-γ–induced chemokine expression can contribute to pathology.

**Figure 5:**
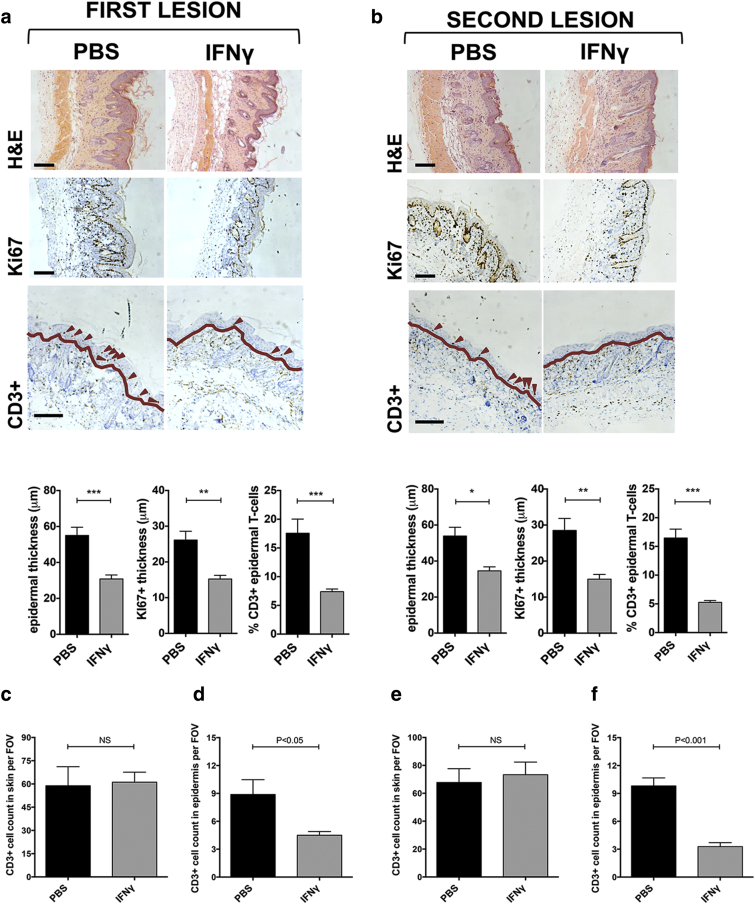
**Histological and immunochemical analysis of primary and secondary IMQ-treated skin sites.** (**a, b**) Representative images of hematoxylin and eosin, Ki67, and CD3^+^ staining and associated quantification of (**a**) initial IMQ-induced lesion with concurrent PBS or systemic IFN-γ (day 5) and (**b**) second IMQ-induced lesion initiated after cessation of initial IMQ/IFN-γ or PBS treatment (day 8) (as in Figure 4b). In CD3^+^ images, dark red arrows = epidermal CD3^+^ T cells and dark red line = dermal-epidermal junction. All scale bars = 100μm. (**c–f**) Total CD3^+^ T-cell numbers in IMQ-induced skin lesion with concurrent PBS or systemic IFN-γ in (**c, d**) first lesion or (**e, f**) second lesion after cessation of systemic PBS or IFN-γ treatment. Numbers shown are for (**c, e**) whole skin and (**d, f**) epidermis. Mean of three separate fields of view/mouse. ^∗^*P* < 0.05, ^∗∗^*P* < 0.01, ^∗∗∗^*P* < 0.005, ^∗∗∗∗^*P* < 0.0001. ACKR2, atypical chemokine receptor 2; FOV, field of view; H&E, hematoxylin and eosin; IMQ, imiquimod; NS, not significant; PASI, Psoriasis Area Severity Index; PBS, phosphate buffered saline.

In agreement with the above data, we found that IFN-γ–mediated ACKR2 up-regulation significantly protected mice against IMQ-induced epidermal thickening, limited epidermal hyperproliferation, and led to a relative exclusion of T cells from the epidermis at the both the primary site and secondary site of IMQ application (Figures 5a and b, and see Supplementary Figure S8 online). When the ear was investigated as a remote site in ACKR2^–/–^ mice (as in Figure 4e), IFN-γ was seen to have no apparent effect, with the IMQ treatment leading to increased dermal/epidermal thickening, enhanced keratinocyte proliferation, and greater epidermal T-cell recruitment compared with IMQ-treated WT ear skin (see Supplementary Figure S9 online). More detailed analysis of the phenotypes of the T cells infiltrating the whole skin (see Supplementary Figure S10a online) and epidermis (see Supplementary Figure 10b) indicated that there were no differences in the overall numbers of each of the phenotypic subsets identified entering the whole skin in response to IMQ in the presence or absence of systemic IFN-γ. In addition, examining the T-cell populations specifically within the epidermal compartment showed the anticipated increase in CD3^+^ T-cell numbers in IMQ-treated ACKR2-deficient mice and further showed that this was heavily skewed toward the CD4^+^ T-cell subset. Differences in epidermal CD3^+^ cell numbers in IFN-γ and vehicle control-treated mice were not simply reflective of differences in total cutaneous T-cell numbers (Figures 5c–f) but instead represented a specific skin-wide positioning of T cells that excluded them from the epidermal compartment. Thus, these data show that localized psoriasiform inflammation, together with elevated circulating IFN-γ, as seen in human psoriasis, protects remote sites from inflammation and that this occurs in an ACKR2-dependent manner.

## Discussion

**Figure 6:**
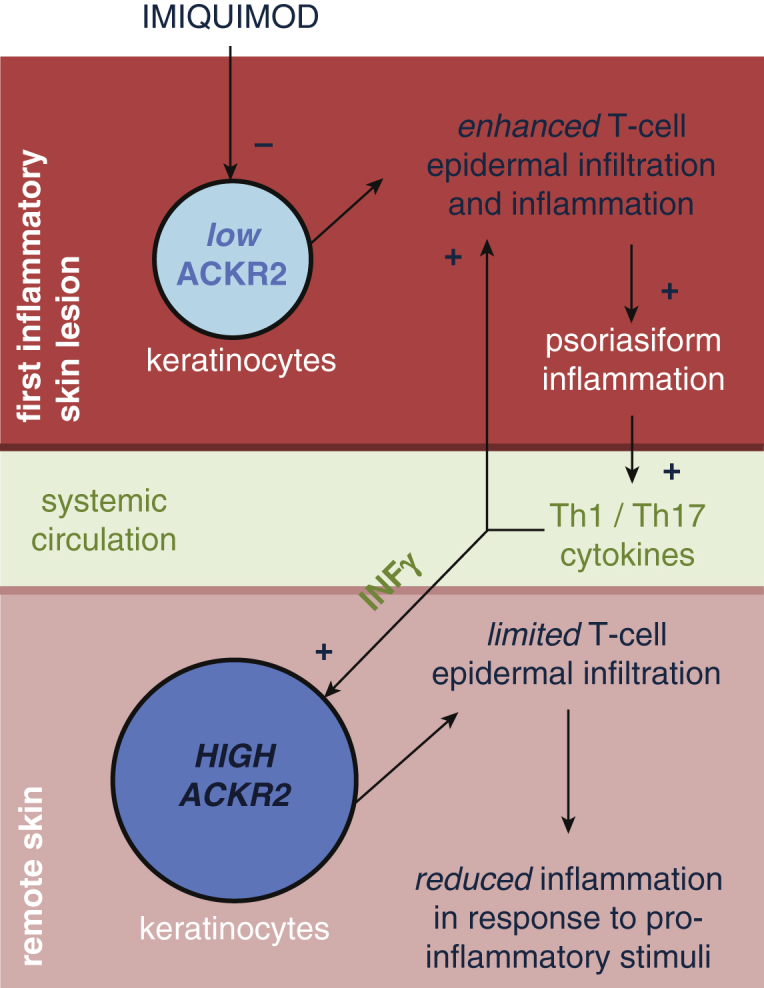
**Schematic model of the main findings of the study.** ACKR2, atypical chemokine receptor 2; Th, T helper.

Mechanistic interactions between localized inflammatory responses and remote tissues are poorly understood. Here we provide evidence that one site of localized inflammation can protect remote tissues from further inflammatory triggers through induced up-regulation of the chemokine scavenging receptor ACKR2. Our model of local and remote ACKR2 function is summarized in Figure 6.

As the main regulators of leukocyte migration, chemokine function is highly regulated. This includes regulation by ACKR2 through its ability to bind, internalize and degrade proinflammatory CC-chemokines (Weber et al., 2004). In psoriasis, ACKR2 is highly up-regulated in clinically unaffected remote skin (Singh et al., 2012). Here, our data show that remote up-regulation of ACKR2 occurs by soluble mediators released from inflammatory lesions and that this limits the spread of inflammation to tissues remote to the original lesion. Our results show that psoriasiform pathology is exaggerated in the absence of ACKR2, with significantly more CD3^+^ T cells localized to the epidermis. Previous work has shown epidermal CD3^+^ T cells to be crucial for the development of psoriatic lesions (Conrad et al., 2007) and CCL5 to be implicated in their accumulation in the epidermis (Collins et al., 2016, Fukuoka et al., 1998). ACKR2 may also contribute to regulating the epidermal (and dermal) accumulation of other inflammatory leukocytes attracted by CC-chemokines, and thus, although our data focus on epidermal T cells, they may have broader relevance for inflammatory cell involvement in pathogenesis. Thus, we provide a mechanism by which a pathology-induced decrease in ACKR2 function can lead to uncontrolled T-cell (and possibly other leukocyte) epidermal entry and, hence, exaggerated inflammatory lesion development.

We found that IMQ-induced inflammation at one site, when supplemented with psoriasis-associated IFN-γ to more closely mimic human disease, was able to modulate induction of new lesions at a second, remote site. Remote sites exhibited increased ACKR2 expression that correlated with restricted epidermal T-cell entry and skin thickening. The ability of systemic IFN-γ to increase ACKR2 expression, in the context of a mouse pathology that does not otherwise involve IFN-γ, suggests that these observations are relevant to both IFN-γ–dependent and –independent inflammatory diseases. In IFN-γ–dependent contexts, if systemic levels are high enough, IFN-γ from the inflamed site may regulate ACKR2 in remote areas to limit the inflammatory spread. In IFN-γ–independent pathologies, systemic administration of IFN-γ can achieve the same end. Our data provide a mechanistic explanation for the previously described therapeutic efficacy of systemic IFN-γ administration in inflammatory diseases including psoriasis (Cannon et al., 1989, Fierlbeck and Rassner, 1990, Fierlbeck et al., 1990, Morhenn et al., 1987, Stevens et al., 1998, Veys et al., 1997). However, the use of IFN-γ for the purpose of up-regulating ACKR2 in humans is likely to be fraught with difficulties, not least because of possible proinflammatory off-target effects and likely injection site reactions (Johnson-Huang et al., 2012). Accordingly, we propose that developing specific inducers of ACKR2 chemokine scavenging provides a new therapeutic approach to treat diseases such as psoriasis and possibly other systemic inflammatory disorders. Remote sites were not protected from developing lesions by IFN-γ in the absence of ACKR2, confirming that this observed protective effect is dependent on ACKR2. This is, to our knowledge, is the first demonstration that primary lesions can modulate the propensity of other sites to become inflamed and that this occurs in an ACKR2-dependent manner.

Together our data suggest that remotely increasing ACKR2 expression levels limits development of cutaneous inflammatory responses and, therefore, highlight a potential therapeutic role in psoriasis and other inflammatory diseases for agents capable of inducing ACKR2 expression.

## Materials and Methods

### Mouse model of psoriasis

The IMQ mouse model was carried out as described (van der Fits et al., 2009). All mouse experiments were performed under the auspices of a UK Home Office license and with permission from the University of Glasgow Ethics Committee. Six- to 8-week-old WT (from Charles River Laboratories, Elphinstone, UK) and ACKR2-deficient (Jamieson et al., 2005) mice on a C57BL/6J background were used throughout the study. A minimum of six mice were used per group in each of the experiments. IFN-γ–treated mice received murine IFN-γ (R&D Systems, Abingdon, UK) 10,000 or 20,000U either intraperitoneally or subcutaneously twice daily (as indicated in figures). IFN-γ was reconstituted in 0.01% sterile BSA in phosphate buffered saline (Sigma-Aldrich, Dorset, UK). Mice were assessed daily, and skin inflammation was scored using a modified Psoriasis Area Severity Index (PASI) (see Supplementary Figure S2). Ear thickness was measured using digital calipers on day of cull.

All tissues for RNA extraction were stored in RNAlater (Life Technologies, Paisley, UK) for 24 hours at 4 °C. For long-term storage, RNAlater-treated tissues were stored at –80 °C. Tissues for histology and immunohistochemistry were fixed in 10% neutral buffered formaldehyde (Sigma-Aldrich).

### RNA extractions and quantitative PCR

RNA was extracted and purified on RNeasy Micro columns with on-column DNase digestion as per manufacturer’s instructions (Qiagen, Manchester, UK). Quantitative PCR was performed as described in the Supplementary Materials online.

### Sections and immunohistochemistry

For hematoxylin and eosin, Ki67, and CD3^+^ staining, paraffin-embedded fixed skin was cut to 2-μm thickness. Heat-induced antigen retrieval was performed in pH 6 sodium citrate buffer for 100 seconds at 125 °C under pressure. Polyclonal rabbit anti-human CD3 (1:100 dilution) and polyclonal rabbit anti-Ki67 (1:400) (both Dako, Carpinteria, CA), or negative controls, were used and counterstained with hematoxylin.

### T-cell and keratinocyte growth and stimulation and generation of human skin equivalents

Details are provided in the Supplementary Materials.

### Migration assay

Details are provided in the Supplementary Materials.

### Analysis of human plasma

Details are provided in the Supplementary Materials.

### Dermal-epidermal splitting

Full-thickness mouse skin was submerged in 0.025-mmol/L EDTA (LifeTechnologies, Paisley, UK) at 37 °C, after which the epidermal layer was curetted off, leaving the dermis intact. Both skin components were immediately lysed in Qiazol, using a Qiagen TissueLyser LT (Qiagen, Manchester, UK).

### Statistics

Student *t* test, Mann-Whitney *U* tests, one-way analysis of variance, two-way analysis of variance, and correlation tests were performed in Prism Version 7.0 (GraphPad Software Inc., San Diego, CA), with multiple comparisons tests as appropriate. Vector data were assessed by the Rayleigh test for vector data in chemotaxis plugin in ImageJ (available at http://imagej.nih.gov/ij/). *P* < 0.05 was deemed significant. ^∗^*P* < 0.05, ^∗∗^*P* < 0.01, ^∗∗∗^*P* < 0.005, ^∗∗∗∗^*P* < 0.0001. All data are *n* ≥ 3 and given as mean ± standard error of the mean unless otherwise stated.

## Conflict of Interest

The authors state no conflict of interest.

## References

[bib1] Bachelerie F., Ben-Baruch A., Burkhardt A.M., Combadiere C., Farber J.M., Graham G.J. (2014). International Union of Pharmacology. LXXXIX. Update on the extended family of chemokine receptors and introducing a new nomenclature for atypical chemokine receptors. Pharmacol Rev.

[bib2] Bachelerie F., Graham G.J., Locati M., Mantovani A., Murphy P.M., Nibbs R. (2014). New nomenclature for atypical chemokine receptors. Nat Immunol.

[bib3] Baldwin H.M., Pallas K., King V., Jamieson T., McKimmie C.S., Nibbs R.J.B. (2013). Microarray analyses demonstrate the involvement of Type I Interferons in psoriasiform pathology development in D6 deficient mice. J Biol Chem.

[bib4] Bazzan E., Saetta M., Turato G., Borroni E.M., Cancellieri C., Baraldo S. (2012). Expression of the atypical chemokine receptor D6 in human alveolar macrophages in chronic obstructive pulmonary disease. Chest.

[bib5] Borroni E.M., Cancellieri C., Vacchini A., Benureau Y., Lagane B., Bachelerie F. (2013). Beta-arrestin-dependent activation of the cofilin pathway is required for the scavenging activity of the atypical chemokine receptor D6. Sci Signal.

[bib6] Cannon G.W., Pincus S.H., Emkey R.D., Denes A., Cohen S.A., Wolfe F. (1989). Double-blind trial of recombinant gamma-interferon versus placebo in the treatment of rheumatoid-arthritis. Arthritis Rheum.

[bib7] Cochain C., Auvynet C., Poupel L., Vilar J., Dumeau E., Richart A. (2012). The chemokine decoy receptor D6 prevents excessive inflammation and adverse ventricular remodeling after myocardial infarction. Arterioscler Thromb Vasc Biol.

[bib8] Codullo V., Baldwin H.M., Singh M.D., Fraser A.R., Wilson C., Gilmour A. (2011). An investigation of the inflammatory cytokine and chemokine network in systemic sclerosis. Ann Rheum Dis.

[bib9] Collins N., Jiang X., Zaid A., Macleod B.L., Li J., Park C.O. (2016). Skin CD4+ memory T cells exhibit combined cluster-mediated retention and equilibration with the circulation. Nat Commun.

[bib10] Conrad C., Boyman O., Tonel G., Tun-Kyi A., Laggner U., de Fougerolles A. (2007). alpha(1)beta(1) integrin is crucial for accumulation of epidermal T cells and the development of psoriasis. Nat Med.

[bib11] Fierlbeck G., Rassner G. (1990). Treatment of psoriasis and psoritic-arthirits with interferon-gamma. J Invest Dermatol.

[bib12] Fierlbeck G., Rassner G., Muller C. (1990). Psoriasis induced at the injection site of recombinant interferon-gamma—results of immunologic investigations. Arch Dermatol.

[bib13] Fukuoka M., Ogino Y., Sato H., Ohta T., Komoriya K., Nishioka K., Katayama I. (1998). RANTES expression in psoriatic skin, and regulation of RANTES and IL-8 production in cultured epidermal keratinocytes by active vitamin D3 (tacalcitol). Br J Dermatol.

[bib14] Graham G.J., Locati M. (2013). Regulation of the immune and inflammatory responses by the 'atypical' chemokine receptor D6. J Pathol.

[bib15] Graham G.J., Locati M., Mantovani A., Rot A., Thelen M. (2012). The biochemistry and biology of the atypical chemokine receptors. Immunol Lett.

[bib16] Harrington L.E., Hatton R.D., Mangan P.R., Turner H., Murphy T.L., Murphy K.M. (2005). Interleukin 17-producing CD4(+) effector T cells develop via a lineage distinct from the T helper type 1 and 2 lineages. Nat Immunol.

[bib17] Jamieson T., Cook D.N., Nibbs R.J., Rot A., Nixon C., McLean P. (2005). The chemokine receptor D6 limits the inflammatory response in vivo. Nat Immunol.

[bib18] Johnson-Huang L.M., Suarez-Farinas M., Pierson K.C., Fuentes-Duculan J., Cueto I., Lentini T. (2012). A single intradermal injection of IFN-[gamma] induces an inflammatory state in both non-lesional psoriatic and healthy skin. J Invest Dermatol.

[bib20] Lowes M.A., Suarez-Farinas M., Krueger J.G. (2014). Immunology of psoriasis. Ann Rev Immunol.

[bib21] Madigan J., Freeman D.J., Menzies F., Forrow S., Nelson S.M., Young A. (2010). Chemokine scavenger D6 is expressed by trophoblasts and aids the survival of mouse embryos transferred into allogeneic recipients. J Immunol.

[bib22] Makino Y., Cook D.N., Smithies O., Hwang O.Y., Neilson E.G., Turka L.A. (2002). Impaired T cell function in RANTES-deficient mice. Clin Immunol.

[bib25] Morhenn V.B., Pregersonrodan K., Mullen R.H., Wood G.S., Nickoloff B.J., Sherwin S.A. (1987). Use of recombinant interferon gamma administered intramuscularly for the treatment of psoraisis. Arch Dermatol.

[bib26] Nibbs R.J., Gilchrist D.S., King V., Ferra A., Forrow S., Hunter K.D. (2007). The atypical chemokine receptor D6 suppresses the development of chemically induced skin tumors. J Clin Invest.

[bib27] Nibbs R.J.B., Graham G.J. (2013). Immune regulation by atypical chemokine receptors. Nat Rev Immunol.

[bib28] Park H., Li Z.X., Yang X.O., Chang S.H., Nurieva R., Wang Y.H. (2005). A distinct lineage of CD4 T cells regulates tissue inflammation by producing interleukin 17. Nat Immunol.

[bib29] Rot A., von Andrian U.H. (2004). Chemokines in innate and adaptive host defense: basic chemokinese grammar for immune cells. Annu Rev Immunol.

[bib30] Singh M.D., King V., Baldwin H., Burden D., Thorrat A., Holmes S. (2012). Elevated expression of the chemokine-scavenging receptor D6 is associated with impaired lesion development in psoriasis. Am J Pathol.

[bib31] Stevens S.R., Hanifin J.M., Hamilton T., Tofte S.J., Cooper K.D. (1998). Long-term effectiveness and safety of recombinant human interferon gamma therapy for atopic dermatitis despite unchanged serum IgE levels. Arch Dermatol.

[bib32] van den Bogaard E.H., Tjabringa G.S., Joosten I., Vonk-Bergers M., van Rijssen E., Tijssen H.J. (2014). Crosstalk between keratinocytes and T cells in a 3D microenvironment: a model to study inflammatory skin diseases. J Invest Dermatol.

[bib33] van der Fits L., Mourits S., Voerman J.S.A., Kant M., Boon L., Laman J.D. (2009). Imiquimod-induced psoriasis-like skin inflammation in mice is mediated via the IL-23/IL-17 axis. J Immunol.

[bib34] Veys E.M., Menkes C.J., Emery P. (1997). A randomized, double-blind study comparing twenty-four-week treatment with recombinant interferon-gamma versus placebo in the treatment of rheumatoid arthritis. Arthrits Rheum.

[bib35] Weber M., Blair E., Simpson C.V., O’Hara M., Blackburn P.E., Rot A. (2004). The chemokine receptor D6 constitutively traffics to and from the cell surface to internalize and degrade chemokines. Mol Biol Cell.

[bib36] Zlotnik A., Yoshie O. (2000). Chemokines: a new classification system and their role in immunity. Immunity.

