# Supplementary material for: Spread of Psoriasiform Inflammation to Remote Tissues Is Restricted by the Atypical Chemokine Receptor ACKR2
Source: J Invest Dermatol. 2017 Jan;137(1):85–94. doi: 10.1016/j.jid.2016.07.039 (PMC5176004; doi:10.1016/j.jid.2016.07.039)
Supplement: Supplementary Data [file mmc1.pdf]

SUPPLEMENTARY INFORMATION

Spread of psoriasiform inflammation to remote tissues is restricted by the atypical chemokine receptor ACKR2. Shams *et al.*

SUPPLEMENTARY FIGURE LEGENDS

Supplementary Figure S1

Enlarged views of (a) H&E and (b) Ki67 stains of WT and ACKR2-deficient (KO) skin after 3 days of IMQ treatment. All scale bars: 100µm.

Supplementary Figure S2: Details of the modified PASI used to assess the extent of psoriasiform pathology in murine skin.

a) The modified PASI used to quantify psoriasiform inflammation on mouse skin on a daily basis. The score ranges from 0=no visible inflammation to 12=severe inflammation.

b) schematic representation of area of mouse flank skin treated with IMQ or vehicle control.

Supplementary Figure S3

Enlarged views of CD3+ staining in WT and ACKR2-deficient (KO) skin after 3 days of IMQ treatment, and resting WT skin. Red arrows indicate epidermal CD3+ T-cells. The red line represents the dermal-epidermal junction. All scale bars: 200µm

Supplementary Figure S4: The design of the T cell migration assay.

a) Diagrammatic representation of our novel migration assay. T-cells and primary human keratinocytes (resting or activated) were added as described in the text. CCL5 was selected as a chemotactic stimulus and added to one side of the collagen gel and a gradient allowed to form through diffusion. T-cell migration towards the CCL5 gradient was imaged by time-lapse photography. Note that the migration assays take place in slides mounted in a horizontal manner on the microscope stage (cellular migration therefore takes place in a horizontal plane in this assay).

b) Manufactured slides holding the layers depicted in (a).

Supplementary Figure S5: Remote tissue histology.

H&E staining of sections from heart and liver from WT and ACKR2-deficient (KO) mice treated on the dorsal skin with vehicle or imiquimod. All scale bars: 400μm.

Supplementary Figure S6: T cell cytokine levels are elevated in psoriatic plasma.

Plasma cytokine levels in healthy controls (*n*=10) versus patients with psoriasis (*n*=50). Significance was determined by Mann-Whitney U test. * $P<0.05$, *** $P<0.005$, *** $P<0.001$.

Supplementary Figure S7: Mouse skin protein expression of IFNγ, CCL5, CCL20 and IL-17.

a-c) Protein concentration of IFNγ, CCL5 and CCL20 in mouse skin on day 5. Mice were given either twice daily IFNγ (20,000 U i.p.) on days 1-4 or vehicle control, to mice treated with either topical IMQ or topical control cream on days 2-4, as measured by ELISA.

d) Absolute CCL20 transcript expression, relative to 18S transcripts, in mouse skin after 3 days of topical application of either control cream or IMQ in WT or ACKR2 deficient (KO) mice.

e) Protein concentration of IL-17 in mouse skin on day 5. Mice treated with IMQ and systemic PBS (vehicle) or IFNγ. Statistics: one-way ANOVA. *$P<0.05$, **$P<0.01$, ***$P<0.005$.

Supplementary Figure S8: Enlarged images of histological and immunohistochemical analysis of primary and secondary IMQ-treated mouse skin (see also Figure 5).

a) The initial IMQ-induced lesion on right mouse flank, with concurrent PBS control or systemic IFNγ at 20,000U per day per mouse and;

b) The second, remote, IMQ-induced lesion initiated following cessation of the initial IMQ/IFNγ or PBS treatment.

In CD3+ stained sections, the dark red arrows indicate CD3+ T-cells in epidermis, with the dark red line representing the dermal-epidermal junction. All scale bars 100μm.

Supplementary Figure S9: Analysis of remote ear skin in ACKR2-/- mice.

H&E staining, Ki67 staining and CD3+ staining of ears of WT mice treated on the ears with IMQ but without systemic IFNγ and ACKR2-deficient mice treated with IMQ on the ear but with attendant systemic IFNγ.

Supplementary Figure S10: T-cell subsets present in IMQ treated mouse skin.

a) Median cell numbers per field-of-view of whole skin (x200 magnification) after 3 days of treatment with IMQ (±systemic IFNγ 20,000 U daily). (Statistics: one-way ANOVA with Tukey's multiple comparisons test). Quantifications carried out using 6 FOV per skin sample. * $P<0.05$, ** $P<0.01$, *** $P<0.005$, **** $P<0.0001$.

b) Median cell numbers per field-of-view of epidermis (x200 magnification) after 3 days of treatment with IMQ (±systemic IFNγ 20,000 U daily). (Statistics: multiple t-test with Holm-Sidak multiple comparison correction. ** = 0.005).

Quantifications carried out using 6 FOV per skin sample.

Supplementary Figure S1

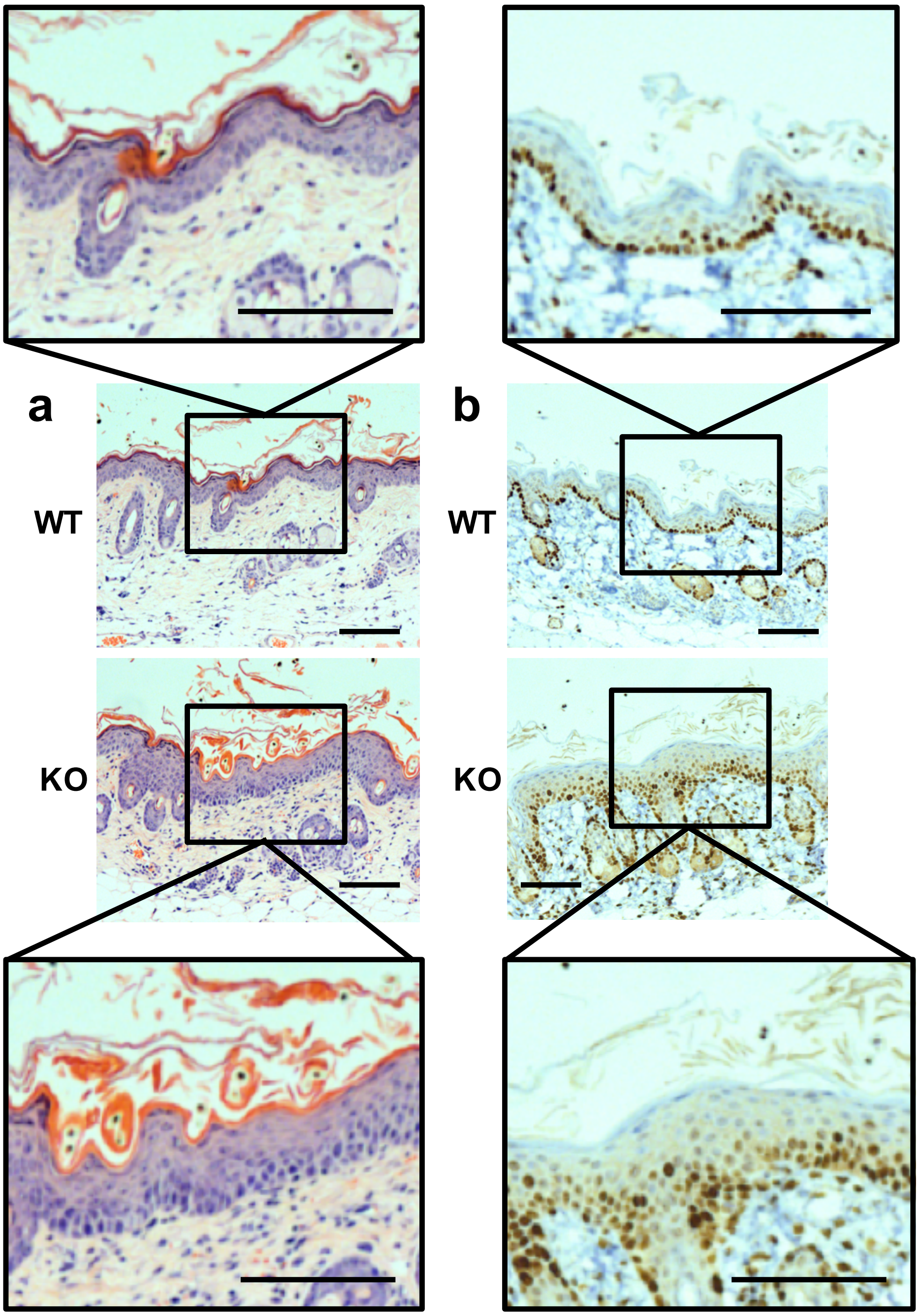

Supplementary Figure S2

a

parameter	score	criteria
erythema	0	normal
	1	pink
	2	red
	3	dark red
	4	very dark red (changing to purple)
scaling	0	normal
	1	fine scale covering part of the lesion
	2	fine scaling covering most of the lesion
	3	rough scaling covering most of the lesion
	4	very rough, thick scaling covering most of the lesion
skin thickness	0	normal
	1	some ridging of skin but dependent on posture
	2	some ridging of skin noticeable when moving
	3	ridging is as need as it is wide and noticeable when moving
	4	very deep ridging noticeable when skin is stretched
**total possible score**	12	sum of all parameters

b

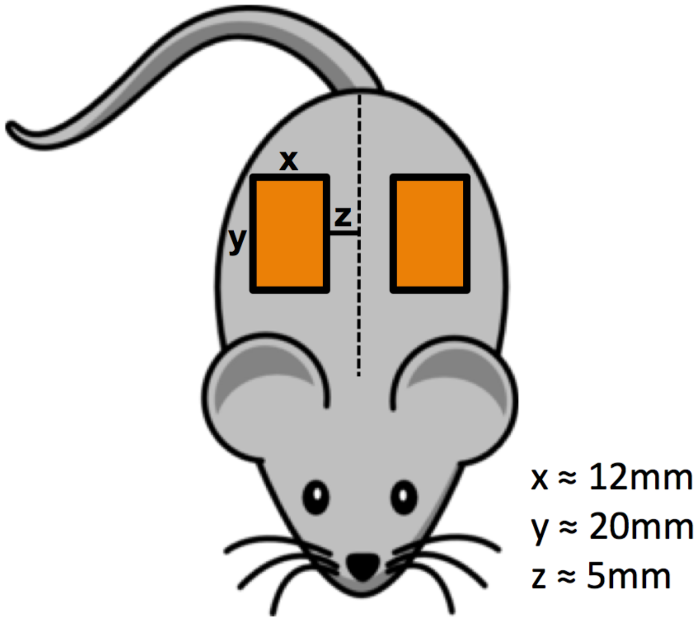

Supplementary Figure S3

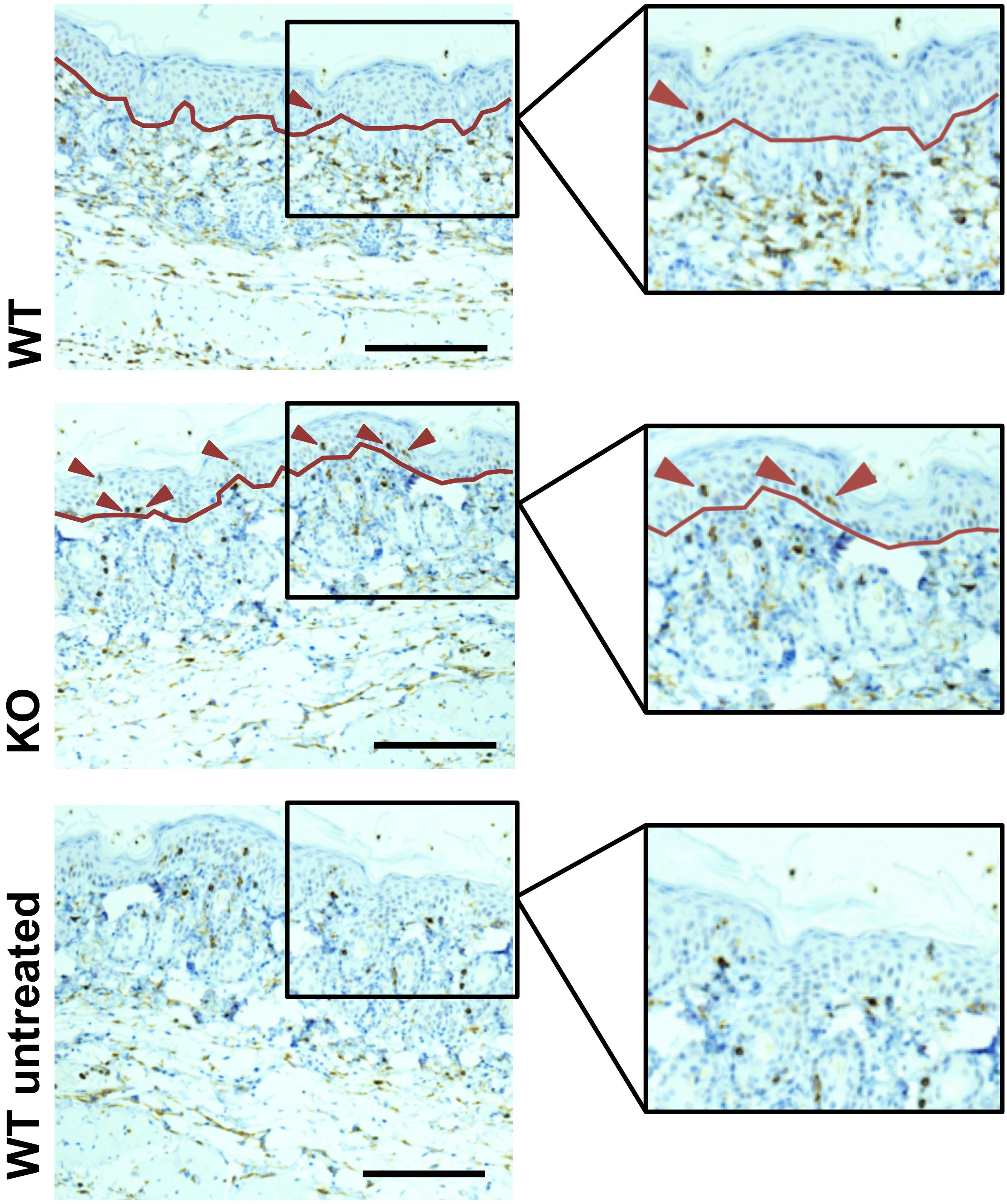

Supplementary Figure S4

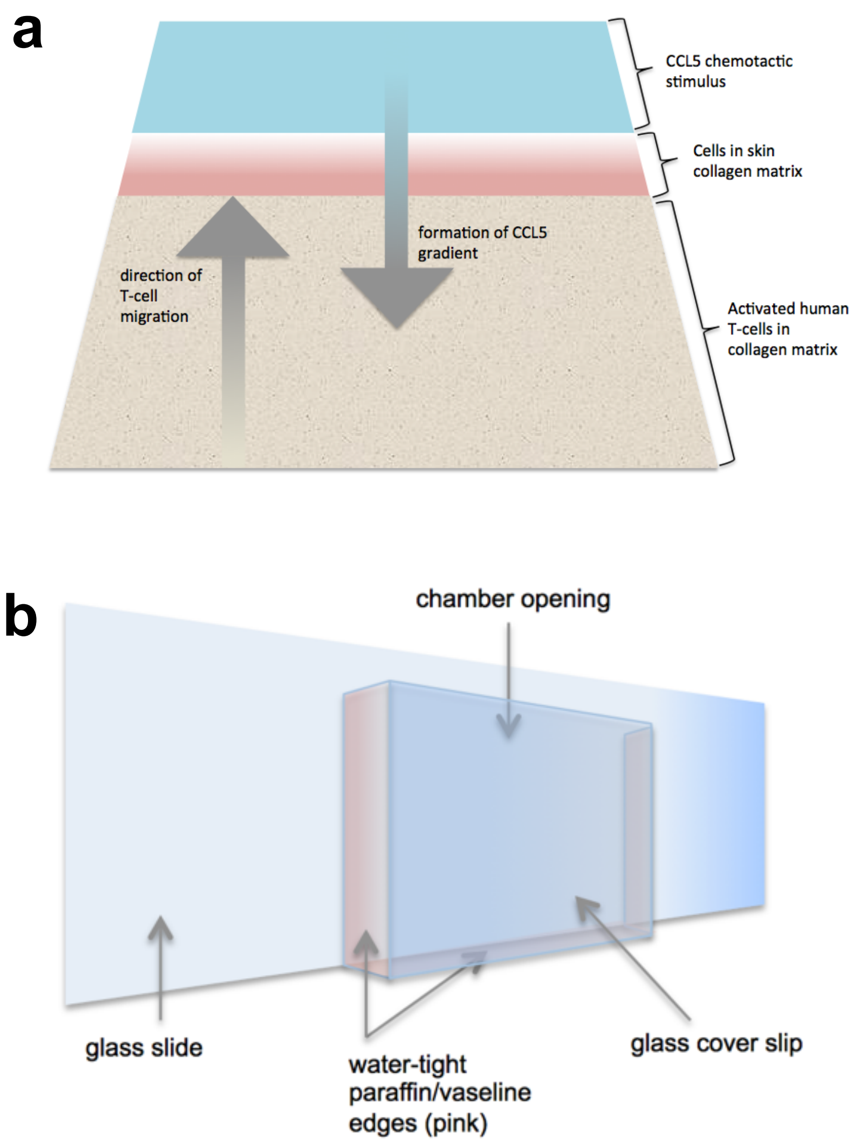

Supplementary Figure S5

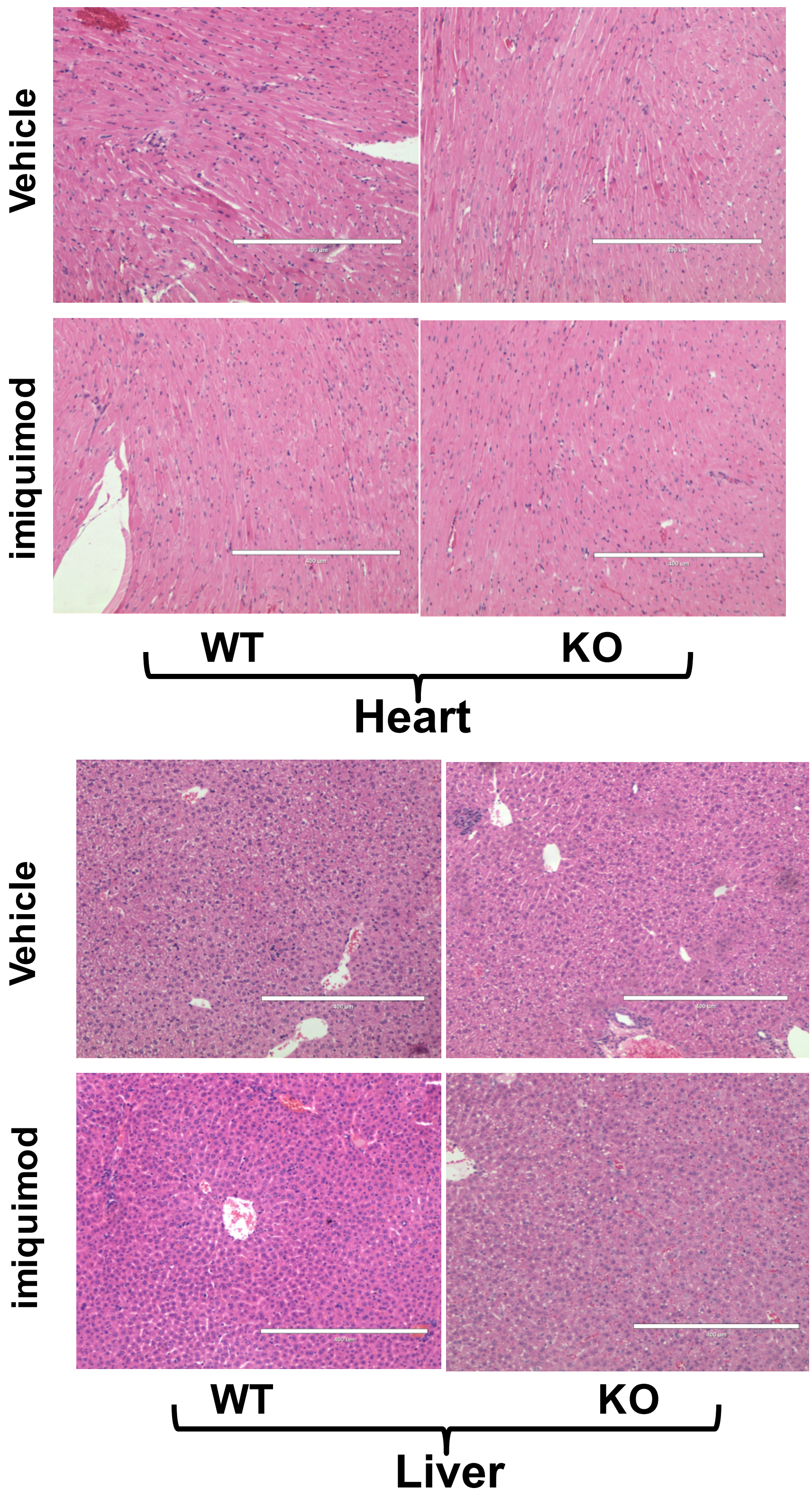

Supplementary Figure S6

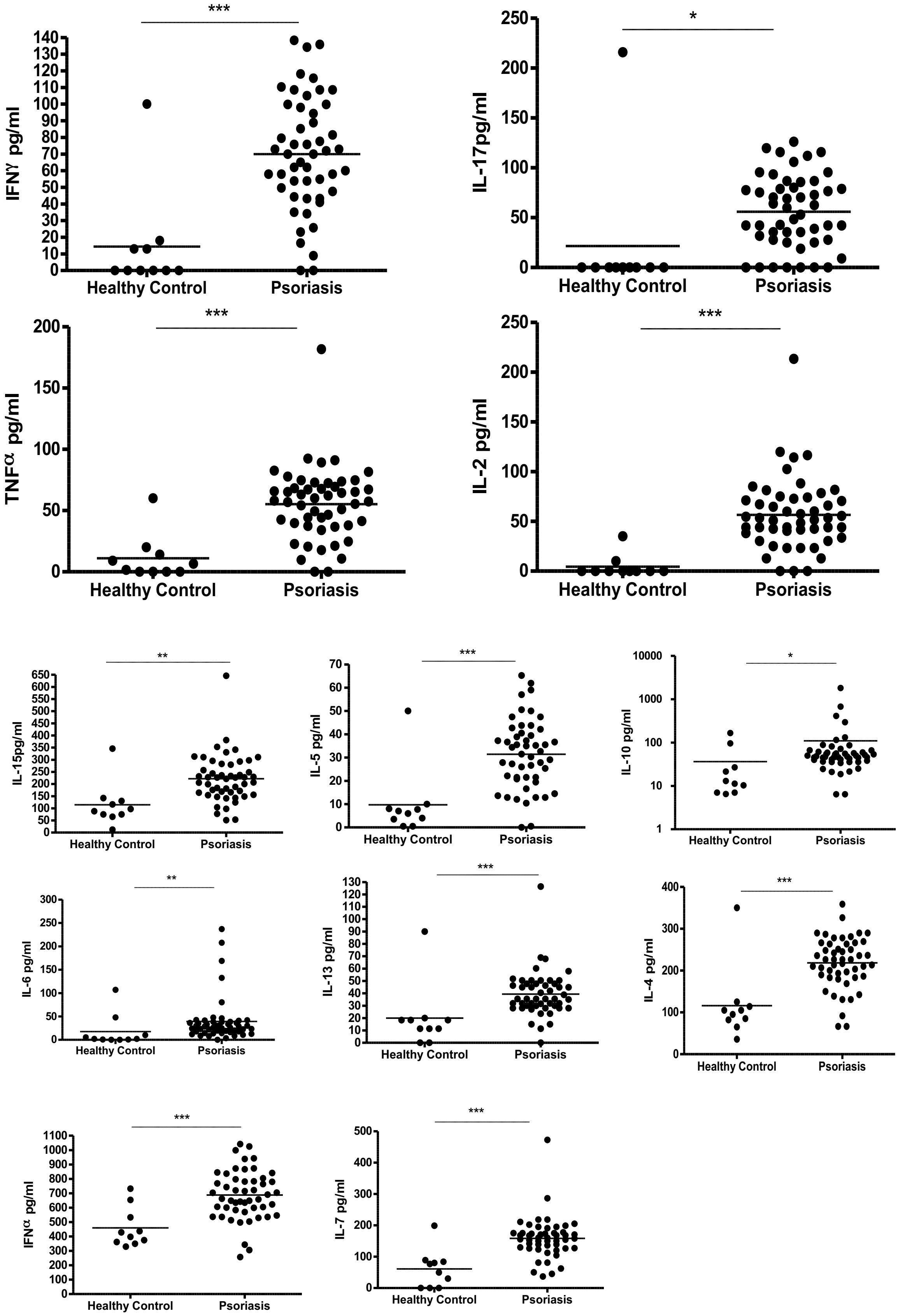

Supplementary Figure S7

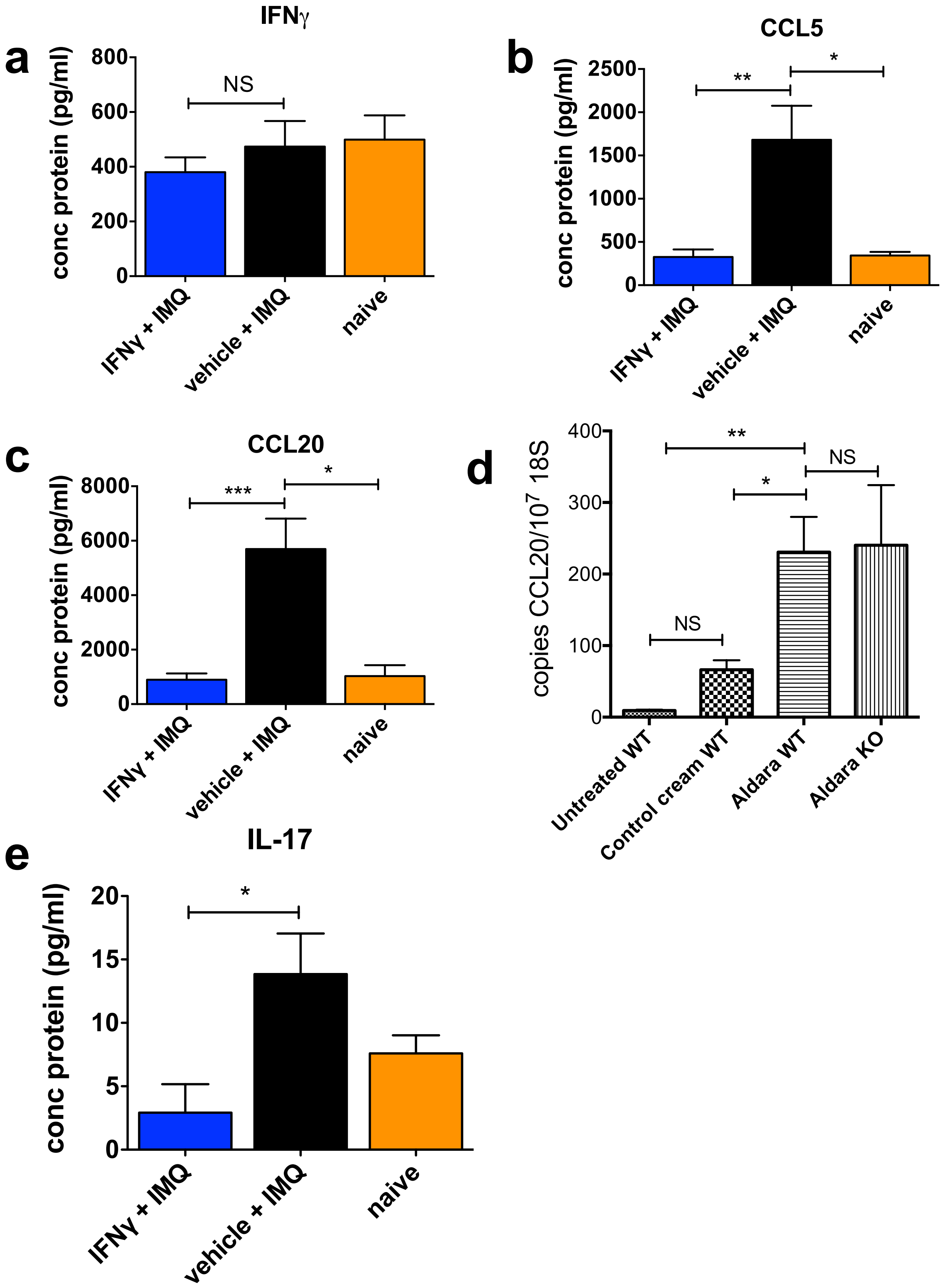

Supplementary Figure S8

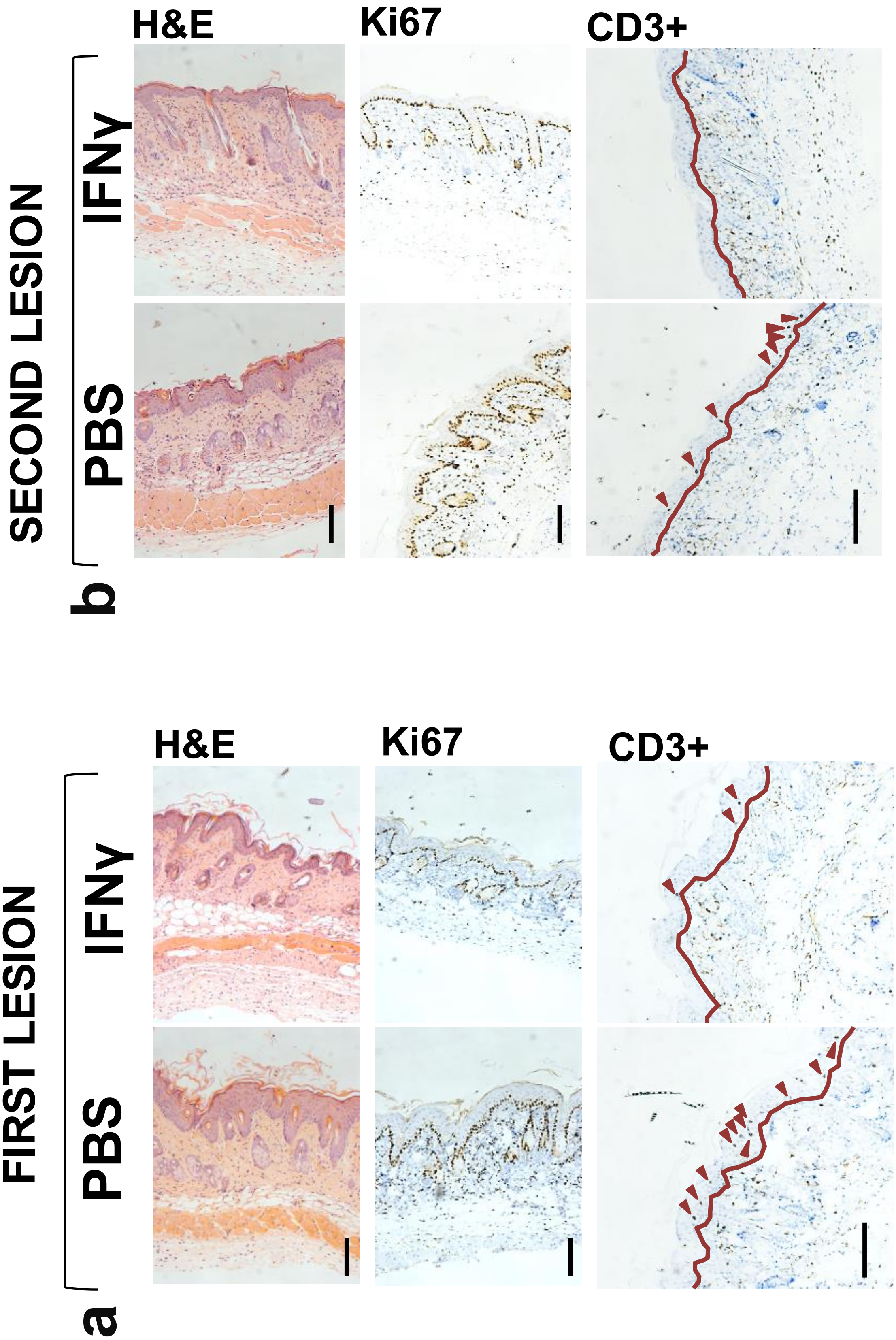

Supplementary Figure S9

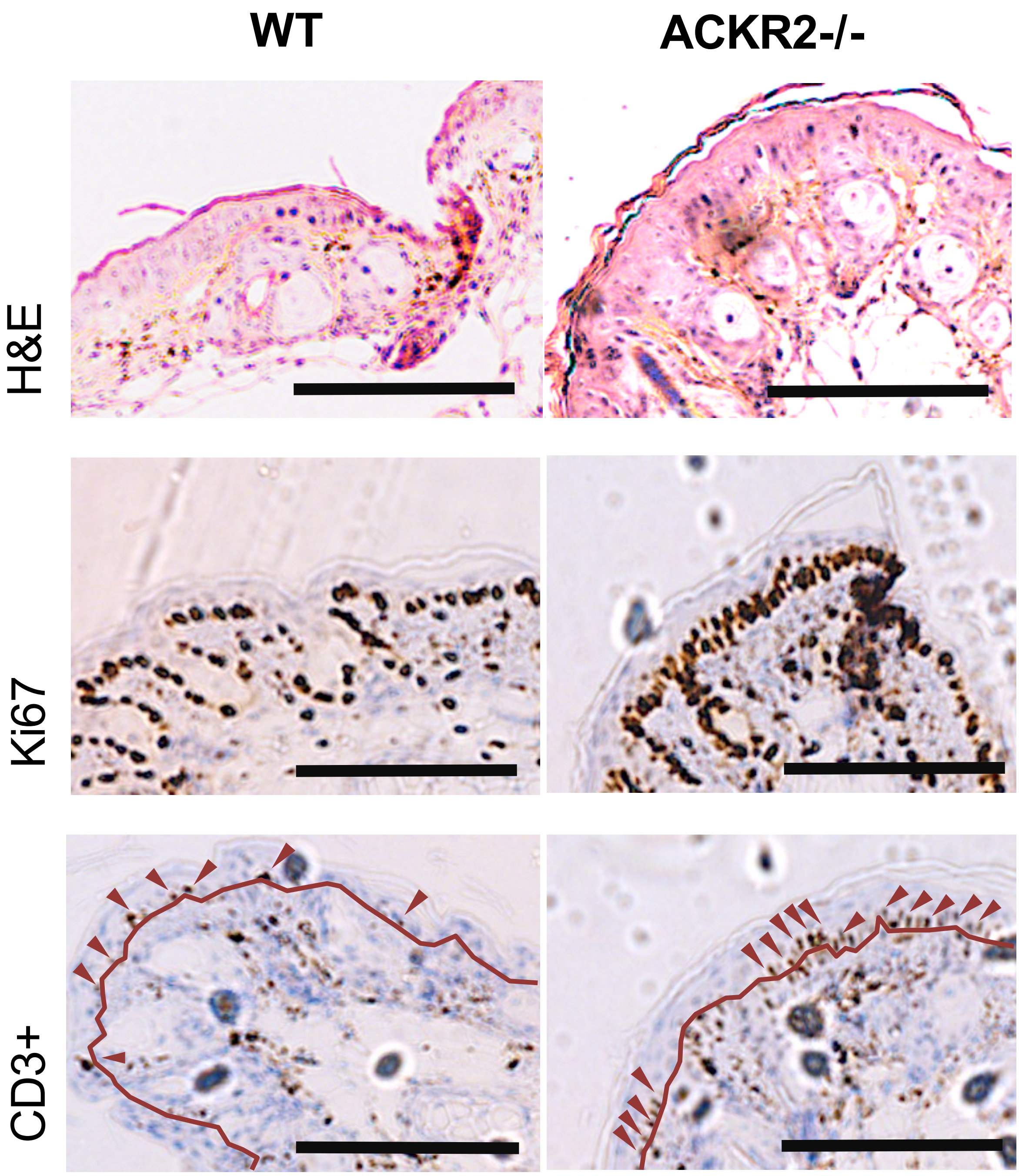

Supplementary Figure S10

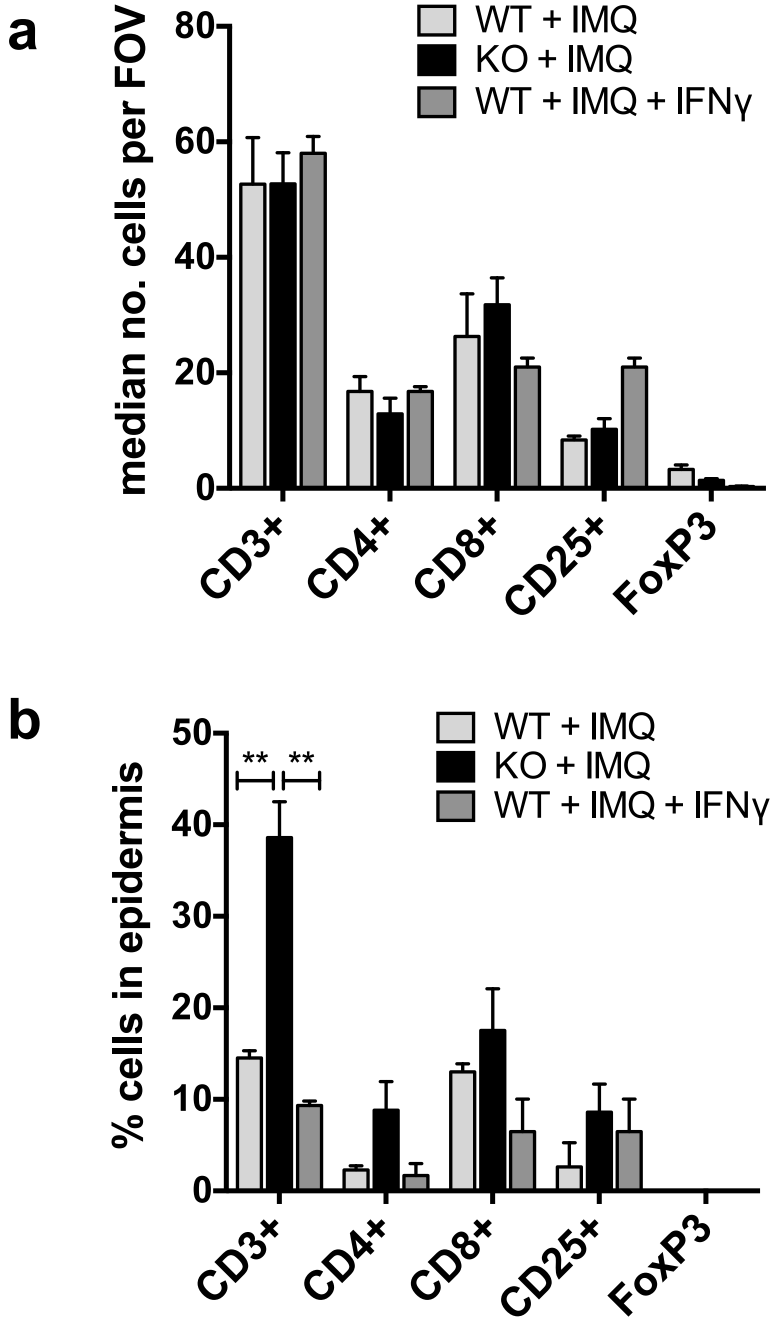

SUPPLEMENTARY MATERIALS AND METHODS

Luminex analysis of human plasma.

Patients for peripheral blood sample collection were recruited from the Department of Dermatology, Western Infirmary Glasgow. All human samples were collected and analysed following, and in accordance with, all necessary approvals from local and regional ethical review boards. Luminex multiplex kits were obtained from Invitrogen (Cytokine Human 30-Plex Panel, LifeTechnologies, Paisley, UK) and plasma analysed, as per manufacturer's instructions.

RNA extractions and Quantitative PCR

RNA was extracted and purified on RNeasy Micro columns, or miRneasy columns (Qiagen, Manchester, UK) with on-column DNAse digestion as per manufacturer's instructions. Whole tissue samples were homogenized in Qiazol with stainless steel beads using a TissueLyser LT (Qiagen). 1 μg total RNA was reverse transcribed using nanoscript RT or RT2 kits as per manufacturer's instructions (PrimerDesign). Gene transcripts were quantified by quantitative PCR analysis using Perfecta SYBR Green master mix as per manufacturer's instructions (Quanta). Samples were analyzed on a 384-well Applied Biosystems 7900HT platform (LifeTechnologies). ACKR2 transcript levels were normalized to human TATA Binding Protein (TBP) for all human transcript analyses and to 18S for all murine transcript analyses, except RPLP0 which was used for the epidermal equivalent studies (van den Bogaard *et al.*, 2014). The sequences for the Q-PCR primers were as previously reported (McKimmie *et al.*, 2008; McKimmie *et al.*, 2013; Singh *et al.*, 2012) except for:

Murine Involucrin - 5'GCTTCAAGGAAACAGCAGCT3' and 5'CTGAGGTTGGGATTGGGGTC3'.

Migration assay

Migration chambers were constructed on sterile glass slides, through suspending a sterile 16x16mm cover slip 1mm over the slide using a 1:5 mix of paraffin and Vaseline on three sides of the cover slip (see Supplementary Figure 5). 500,000 activated T-cells were suspended in a 300µl collagen matrix (bovine skin collagen, MEM and sodium bicarbonate, all Sigma) and the chamber filled half-way and allowed to set for 1 hour in a humidified incubator at 37°C with 5% CO_2. A 1mm acellular layer was subsequently placed on the T-cell containing matrix and allowed to set. A further 1mm layer was subsequently added on top of this layer, containing 10% keratinocytes by volume and again allowed to set. All cells were washed twice in serum-free medium before use, and re-suspended in serum-free medium. 160ng/ml CCL5 (PeproTech) reconstituted in keratinocyte medium was layered on top of the cell matrix, and the top of the chamber sealed. The slide was kept flat at 37°C and T-cell migration captured in one focal plane at 2 frames per minute for 3 hours immediately upon addition of CCL5 (Zeiss AxioImager M2 epifluorescence microscope (Germany) with Zeiss, ZEN Software). Image analysis was performed in ImageJ, using Manual Tracking and Chemotaxis plugins (http://imagej.nih.gov/ij/).

T-cell isolation and stimulation

Human T-cells were grown from CD14-depleted human buffy coats from healthy donors, and stimulated with Concanavalin A (5ng/ml, Sigma), and grown in RPMI (LifeTechnologies) with 5% human AB serum and gentamicin (Sigma) in the presence of IL-2 (20 units/ml, Peprotech) from day 4. Cells were purified on Ficoll-Paque after 8 days (GE Healthcare) and grown in the presence of IL-2 alone for 4 days, before being activated using

CD2/CD3/CD28 beads as per manufacturer's instructions at a 1:2 bead:cell ratio (Miltenyi Biotec) for 24 hours prior to the activated supernatant being removed for downstream applications. Where T-cell supernatant was to be used for keratinocyte stimulation, T-cells were grown in serum-free keratinocyte medium KCGM2 with added supplements during the 24-hour activation period (PromoCell). T-cells used for migration assays were similarly activated albeit at a 1:4 bead:cell ratio for 48 hours prior to use.

Primary human cell culture

Primary human epidermal keratinocytes (phKC) and lymphatic endothelial cells (LEC) from healthy donors were purchased from PromoCell (Heidelberg) and grown in serum free Keratinocyte Growth Medium 2 with added supplements, calcium 0.06mM, and Endothelial Growth Medium MV2 respectively, as per manufacturer's instruction. Cells were grown in the presence of penicillin/streptomycin and gentamicin (Sigma). Cells were used at passage 3 or 4. In stimulation experiments, the following agents were used at concentrations as indicated in the Figures: recombinant human IFNγ (Peprotech), anti human IFNγ monoclonal antibody (R&D Systems) and tissue culture supernatant of activated human T-cells. All cells were grown in a humidified incubator at 37°C with 5% CO_2.

Generation of human inflammatory skin equivalents

Methodology for generating skin equivalents is as previously described (van den Bogaard *et al.*, 2014). To study the effect of ciclosporin A (400ng/ml; Novartis Pharmaceuticals Corporation, Arnhem, the Netherlands), and all-trans retinoic acid (10^{-6} M; Sigma-Aldrich) the drugs were administered two days after T-cell migration into the skin equivalents. CD4+ T-cells were purified from peripheral blood mononuclear cells from healthy blood donors by negative selection as previously described (Koenen *et al.*, 2008) and

depleted for CD25^{high} Treg using CD25 magnetic-activated (MACS) cell sorting beads (Miltenyi-Biotec). T-cells were activated using anti-CD3/CD28-beads (Life Technologies) at a T-cell:bead ratio of 5:1 for 5 hours. Following activation, T-cells were washed in skin equivalent medium and added to the skin equivalent.

REFERENCES

Koenen HJPM, Smeets RL, Vink PM, van Rijssen E, Boots AMH, Joosten I. Human CD25(high)Foxp3(pos) regulatory T cells differentiate into IL-17-producing cells. Blood. 2008;112:2340-52.

McKimmie CS, Fraser AR, Hansell C, Gutierrez L, Philipsen S, Connell L, et al. Hemopoietic cell expression of the chemokine decoy receptor D6 is dynamic and regulated by GATA1. J Immunol. 2008;181:8171-81.

McKimmie CS, Singh MD, Hewit K, Lopez-Franco O, Le Brocq M, Rose-John S, et al. An analysis of the function and expression of D6 on lymphatic endothelial cells. Blood. 2013; 121:3768-77.

van den Bogaard EH, Tjabringa GS, Joosten I, Vonk-Bergers M, van Rijssen E, Tijssen HJ, et al. Crosstalk between Keratinocytes and T Cells in a 3D Microenvironment: A Model to Study Inflammatory Skin Diseases. Journal of Investigative Dermatology. 2014;134:719-27.
